# Syphilis in the HIV Era

**DOI:** 10.3201/eid1008.031107

**Published:** 2004-08

**Authors:** Sigall Kassutto, John P. Doweiko

**Affiliations:** * Beth Israel Deaconess Medical Center, Boston, Massachusetts, USA

**Keywords:** syphilis, epidemiology, Tullio phenomenon, HIV, MSM, dispatch

## Abstract

The incidence of syphilis has consistently increased from 2000 to 2002. We report a case of acquired syphilis with symptoms of Tullio phenomenon in a patient concurrently diagnosed with HIV infection. The resurgence of syphilis in HIV-positive groups at high risk has public health implications for prevention of both diseases.

## The Case

A 35-year-old man with an unremarkable medical history sought treatment for headaches, hearing loss, and night sweats. The headaches were occipital and bilateral and had started 3 months earlier. They came on as the day progressed and were neither positional nor associated with nausea, vomiting, or visual changes. Three weeks before arriving at the hospital, he noted a sense of "fullness" in his ears; he said that spoken voices sounded muffled, and he had difficulty hearing telephone conversations. When someone at the restaurant where he worked dropped a dish, he heard that sound clearly and reported that it was almost painful, causing him to become dizzy. He otherwise denied ear pain or recent trauma. His appetite was good, and he had not lost weight. Although he reported no fever, he did report night sweats for several weeks. He had no rash, diarrhea, abdominal pain, chest pain, shortness of breath, joint complaints, or dysuria. The patient attributed his headaches to stress. He also said that he was exposed to dust at his workplace because of remodeling.

The patient was taking no regular medications and had no drug allergies. He had quit smoking 7 months earlier and drank 10–12 beers per week. He reported no history of intravenous drug use. He lived in New Hampshire, had no pets, and worked as part owner of a restaurant. Exposures included multiple male and female sexual partners with inconsistent condom use, and he had acquired several tattoos 8 months earlier while traveling in Spain and Italy. He had no known tuberculosis exposure. An HIV antibody test had been negative 18 months earlier.

On physical examination, he appeared healthy but anxious. Temperature was 36.7°C, blood pressure 128/84 mm Hg, pulse 80, respirations 16/minute. Sclerae were anicteric, and his pupils reacted to direct and consensual testing and responded normally to accommodation. Funduscopic examination showed no retinal abnormalities. The left tympanic membrane was retracted; both sides demonstrated a small effusion. No vesicles were seen. Bedside testing showed that his hearing was diminished to low volume sounds, but he was able to hear loud sounds, which he found painful. When the examiner clapped his hands loudly a few feet from the patient's ear, the patient exhibited nystagmus. His sinuses were not tender, and the oral mucosa had no lesions. The patient's neck was supple; a 1.5-cm, nontender lymph node was palpated in the left upper anterior cervical chain. No other lymphadenopathy was noted. The remainder of the examination was unremarkable, with negative Romberg test; normal gait; and normal motor, sensory, and reflex performance.

Laboratory evaluation (Table) showed low hematocrit and normal renal and liver function. HIV enzyme-linked immunosorbent assay and confirmatory test were positive. Rapid plasma reagin (RPR) was positive at a titer of 1:128 and was confirmed by a fluorescent treponemal antibody test. Subsequent studies showed a CD4 receptor–positive T-cell (CD4) count of 899/mL and an HIV viral load of 878 copies/mL. A lumbar puncture showed protein 33 mg/dL, glucose 78 mg/dL, 9 erythrocytes/µL, and 8 leukocytes/µL (100% lymphocytes). Cerebrospinal fluid venereal disease research laboratory test (CSF-VDRL) results were negative.

**Table:** Selected laboratory test results 1 month after therapy for neurosyphilis in HIV-positive patient with neurosyphilis^a^

Laboratory test	Result
Complete blood count	
Leukocytes	5,700/µL
Hematocrit	36%
Platelets	295,000/µL
Electrolytes and renal function tests	Within normal limits
Liver function tests	Within normal limits
Cerebrospinal fluid evaluation	
Leukocytes	8 cells/µL
Differential	100% lymphocytes
Erythrocytes	9 cells/µL
Protein	33 mg/dL
Glucose	78 mg/dL
CSF-VDRL	Negative
HIV-1 testing	
ELISA + WB	Positive
CD4 count	899 cells/µL
Viral load (RT-PCR)	878 copies/mL
Rapid plasma reagin testing	
Baseline	1:128
1 month posttreatment	1:32
3 months posttreatment	Nonreactive

^a^CSF-VDRL, cerebrospinal fluid venereal disease research laboratory test; ELISA, enzyme-linked immunosorbent assay; WB, Western blot; RT-PCR, reverse transcription–polymerase chain reaction.

The patient was started on 24 mU of IV penicillin per day for 14 days. At the end of this period, he reported notable improvement in his headaches, hearing loss, and vertiginous symptoms. One month after completing treatment, his RPR titer was 1:32; 3 months after he completed treatment, it was nonreactive.

## Conclusions

The course of syphilis in an HIV-positive patient may be altered from the natural history of the disease in HIV-negative patients. An increased frequency of ocular disease, multiple and slower resolving primary chancres, and a higher titer RPR have been reported (1–6). In addition, delay or failure of titer decline after treatment, predilection for developing the Jarisch-Herxheimer reaction, and clinical relapse have also been described in HIV infection (1,4,7,8).

*Treponema pallidum* is thought to invade the central nervous system in 25% of patients with syphilis, irrespective of HIV status (9). Most of these persons successfully clear the infection (10), but other patients harbor the spirochete and remain at risk for sequelae of neurosyphilis. Syphilitic meningitis, meningovascular disease (often occurring with stroke), labyrinthitis, or cranial nerve palsies, as seen in this case, can be early findings.

The Tullio phenomenon is vestibular hypersensitivity to sound. In this condition, loud sounds or even routine acoustic stimuli can result in vertigo, nystagmus, or nausea and vomiting. The physiologic underpinnings of the Tullio phenomenon were first described in 1929, when Tullio noted that experimentally induced fenestrations in the bony capsule of the lateral semicircular canals of pigeons caused the canals to be sound-responsive, inducing vestibular activation (11,12). Shortly thereafter, Benjamins described a Tullio reaction in a human patient with fistulizing cholesteatoma (13). Today, the term has been generalized to include vestibular activation in response to stimulation by sound of any part of the vestibular apparatus (12).

The Tullio phenomenon is seen in a range of clinical contexts, including congenital deafness, Meniere disease, suppurative middle ear disease, and spirochetal infections, such as syphilis or Lyme disease. Nields et al. describe a woman who had nystagmus and vertigo with routine sounds; running tap water caused her to fall to the floor or retch in pain (14). Watson et al. describe a series of patients with oscillopsia induced by pencil tapping, telephone ringing, or the sound of cutlery falling on the floor. Patients often report a vague sense of ear blockage and an unpleasant awareness of their own voice vibrating in their ear (12).

Symptomatic cranial nerve VIII involvement in this patient prompted treatment for neurosyphilis, despite equivocal (but typical) CSF findings. Interpreting CSF findings in HIV-positive patients with syphilis is challenging because commonly encountered mild lymphocytic pleocytosis may be attributable to HIV. CSF-VDRL is often negative, with a 20%–70% false-negative rate (15).

Optimally managing patients with HIV and syphilis has been debated; to date routine recommendations are the same as for HIV-negative persons (16). Although any patient in whom syphilis is diagnosed and who has neurologic symptoms should undergo lumbar puncture, CSF evaluation should also be considered in asymptomatic patients in whom syphilis is diagnosed and who have an RPR titer >1:32 or a CD4+ count <350, as these markers have been associated with increased risk for neurosyphilis (10). Because of the high rate of relapse that has been reported in some series (4,7,8), close clinical and serologic follow-up is essential. Whether *T. pallidum* eradication is impaired in HIV coinfection remains controversial. A study of 59 patients with neurosyphilis showed that HIV-positive study participants were 2.5 times less likely to normalize CSF-VDRL reactivity than HIV-negative patients. This effect was even more pronounced in patients with CD4+ counts <200 (17). The findings suggest that more intensive therapy for neurosyphilis in immunocompromised patients merits further study.

After an all-time low case-rate of syphilis in 2000, reports of rising trends, specifically in groups at risk, such as men who have sex with men, were reported in 2001 (18). The outbreaks marked a 9.1% overall increased rate of primary and secondary syphilis (19) and were characterized by a high rate of HIV infection. Parallel increases in HIV infection rates were a concern because of the common mechanism of transmission and the increased efficiency of HIV transmission with coexistent genital ulcers (20). In 2001, increases in HIV rates were reported in several states with large urban populations; men who have sex with men accounted for the largest known subgroup at risk in incident adult or adolescent HIV cases (32%) and AIDS cases (44%) (21). New HIV diagnoses increased in 29 states with mandatory reporting from 1999 to 2002, notably by 17% among gay and bisexual men (22). Syphilis incidence also continued to rise in the United States in 2002, with a 12.4% increase since 2001 (19), and the highest trends were estimated to be among men who have sex with men. In San Francisco, primary and secondary syphilis rates increased by >1,000% from 1998 to 2002 among men who have sex with men (23). High-risk behaviors have been documented in this group (24) and factor prominently in syphilis reemergence in the early 21st century.

This case report describes a patient who had Tullio phenomenon as the index symptom of neurosyphilis with previously undiagnosed HIV infection. His RPR titer was 1:128, his CD4 count was preserved, and he responded well clinically and serologically to standard therapy. To our knowledge, this case is the first report of syphilis occurring as Tullio phenomenon in an HIV-positive patient. We suggest that syphilis be considered in patients who have cranial nerve VIII symptoms and an appropriate risk-factor profile. Eradicating treponemes may be limited from sanctuary sites such as the CNS. Because control of syphilis likely depends not only on antimicrobial effect but also on host immune response, routine surveillance is a mainstay of therapy, particularly in patients with HIV infection. Awareness of unusual symptoms of a relatively common disease will benefit not only the patient but also public health efforts in managing both syphilis and HIV infection. Preventive and educational efforts focused on men who have sex with men may prove particularly important in modifying behaviors that foster the growth of both epidemics.
